# Yeast-Based Virus-like Particles as an Emerging Platform for Vaccine Development and Delivery

**DOI:** 10.3390/vaccines11020479

**Published:** 2023-02-18

**Authors:** Vartika Srivastava, Kripa N. Nand, Aijaz Ahmad, Ravinder Kumar

**Affiliations:** 1 Department of Clinical Microbiology and Infectious Diseases, School of Pathology, Faculty of Health Sciences, University of the Witwatersrand, Johannesburg 2193, South Africa; 2 Department of Biological Sciences, Rensselaer Polytechnic Institute, Troy, NY 12180, USA; 3 Infection Control, Charlotte Maxeke Johannesburg Academic Hospital, National Health Laboratory Service, Johannesburg 2193, South Africa

**Keywords:** yeast, virus-like particles (VLPs), subunit, drug delivery, vaccine

## Abstract

Virus-like particles (VLPs) are empty, nanoscale structures morphologically resembling viruses. Internal cavity, noninfectious, and particulate nature with a high density of repeating epitopes, make them an ideal platform for vaccine development and drug delivery. Commercial use of Gardasil-9 and Cervarix showed the usefulness of VLPs in vaccine formulation. Further, chimeric VLPs allow the raising of an immune response against different immunogens and thereby can help reduce the generation of medical or clinical waste. The economically viable production of VLPs significantly impacts their usage, application, and availability. To this end, several hosts have been used and tested. The present review will discuss VLPs produced using different yeasts as fermentation hosts. We also compile a list of studies highlighting the expression and purification of VLPs using a yeast-based platform. We also discuss the advantages of using yeast to generate VLPs over other available systems. Further, the issues or limitations of yeasts for producing VLPs are also summarized. The review also compiles a list of yeast-derived VLP-based vaccines that are presently in public use or in different phases of clinical trials.

## 1. Background of Vaccines

The term vaccine originated from the Latin word “Vacca”, meaning cow. Vaccines are a biological formulation of weakened or killed pathogens (sometimes closely related species of the target pathogen), which, upon administration, help protect an individual against the target disease(s) by imitating an infection. Generally, vaccines are prophylactic except for the vaccine against rabies, which is curative [1]. Primarily, whole microbial pathogens were used in vaccine formulation along with other constituents such as preservatives or stabilizers, adjuvants, or immune boosters [2]. However, the conventional vaccine development strategy is not suitable for combating those diseases where the associated pathogen defies the current microbiological growth procedures on a commercial scale (for example, *Mycobacterium leprae* and species of *Plasmodium*) [3,4].

**Figure 1:**
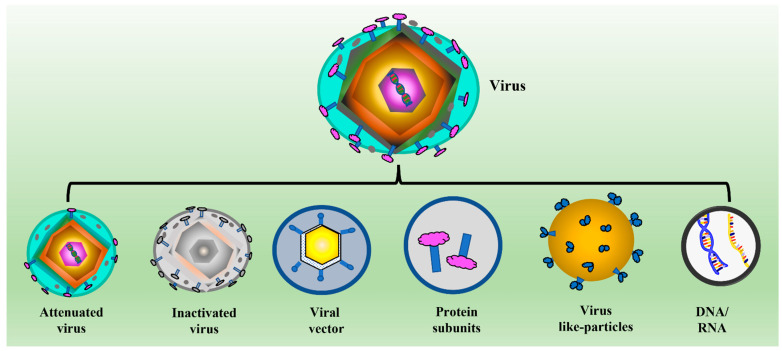
Schematic showing the different strategies of anti-viral vaccine development. The figure shows both conventional (attenuated and killed virus) and newer methods of vaccine development (including subunit, nucleic acid, and VLP-based vaccines).

The arrival of subunit vaccines, which involved only a few components of the pathogen, overcame the loophole associated with the traditional vaccine development regime. Apart from the surface proteins (from bacteria, protozoa, and viruses), subunit vaccines may also include pathogen-specific toxin(s) and polysaccharides [5]. The most recent and dramatic development in vaccine formulation is nucleic acid-based vaccines (which include DNA and mRNA) [6]. These vaccines overcome the issues associated with conventional and subunit vaccine development processes, such as the expression and purification of target proteins and the need to propagate serious pathogens [7,8,9]. Thus, nucleic acid-based vaccines gave a new horizon to the therapeutic regimen in healthcare settings; for instance, mRNA-based vaccines against COVID-19 (manufactured by Moderna and Pfizer) are an advancement over the traditional vaccine development against infectious diseases [10,11,12,13]. Thus, there is continuous improvement in the methods and strategies used in vaccine development and delivery. Due to the arrival of newer methods of vaccine design and development, there is a need to redefine vaccines. Today, vaccines can be redefined as biological preparations containing attenuated or killed pathogens or any component, including peptides, proteins, DNA, mRNA, or polysaccharides, which, upon injection, help prevent an individual from any possible future infection [6]. Although subunit and nucleic acid-based vaccines offer several advantages over conventional methods of vaccine development, they still suffer from issues like the need for a cold chain or deep refrigeration and a short shelf life like other common vaccines [14]. Figure 1 shows the different methods of antiviral vaccine formulation.

Initially, vaccines were used only against infectious diseases (viral or bacterial), but now vaccines are considered the most promising means to combat certain types of cancer. For example, Gardasil-9 (the L1 protein of the *human papillomavirus, or HPV*), is expressed and purified from yeast against *HPV*, whose infection may lead to cervical cancer. Thus, in the contemporary world, vaccines are not just a tool to fight against infectious diseases but are also used to prevent certain types of cancer associated with viral infections [15,16,17]. Apart from papilloma, several other anticancer vaccines are in different stages of clinical trials [18,19,20,21,22].

Vaccine development platforms based on subunits or nucleic acids have actualized the development of vaccines against diseases considered unfeasible a few decades ago (for example, a vaccine against malaria) [23]. Additionally, these approaches have expedited vaccine development programs and made them safer for individuals; the development and application of an mRNA-based vaccine against COVID-19 is the best-suited example for this kind of platform [10,11,12,13]. A continuous cold chain is needed to maintain the efficacy or potency of vaccines. To tackle this, researchers are exploring further advancements to fix the problem associated with the thermolabile nature of vaccines and minimize the requirement of a cold chain, as discussed elsewhere [14,24,25].

After briefly introducing vaccines, the different development platforms used, and their shortcomings, it is now time to focus on one of the emerging platforms in the field of vaccine development. The intended purpose of this review is to discuss the suitability of VLPs as a means of vaccine development and drug delivery, with a particular focus on VLPs derived from yeast. We will also discuss how VLPs are a better platform than purified proteins for triggering immune responses in the host. The present review also highlights the reasons that make yeast the most acceptable model for the expression and purification of VLPs; the review also includes associated concerns and success stories about this concept. We also discuss the major yeast species used to express and purify VLPs. All of this is discussed under separate headings.

## 2. Introduction to Virus-Like Particles

VLPs, ghost viruses, or dummy viruses lacking genetic material are nanostructures first observed in sera samples from hepatitis patients in 1968 [26]. VLPs can exist naturally (in a virally infected host) and can be generated in the laboratory. The size of VLPs may vary from 20 nm to 200 nm or more [27]. The size of VLPs depends on the virus species and viral proteins used for developing these particles [27]. It is essential to mention that VLPs can be formed using the capsid, envelope, or core viral proteins [27]. These particles are usually formed naturally by folding viral proteins under appropriate conditions, including the optimum pH, salt concentration, temperature, and so on [28,29,30]. The VLPs form when monomeric proteins fold into a pentameric form, also called capsomers, which are then assembled to form VLPs [30]. VLPs can exist either non-enveloped, as seen in the case of HPV VLPs, or enveloped with a lipid membrane (eVLPs), such as SARS coronavirus VLPs [31,32]. The shape of VLPs also differs from icosahedral to rod-shaped [33]. These particles may be composed of a single protein or can be a fusion of two different proteins (chimeric VLPs) [34,35].

**Figure 2:**
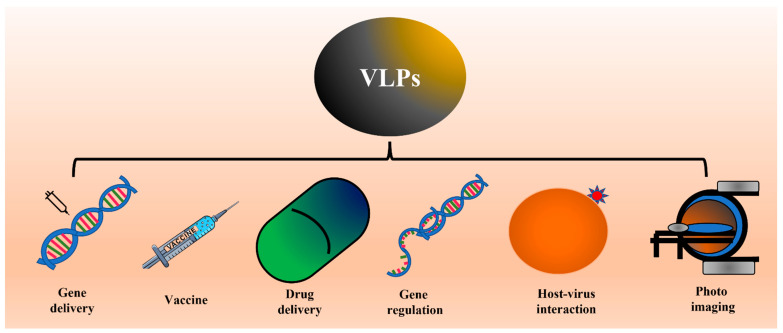
Schematic showing the different applications of VLPs. Several studies have already shown proof of concept for applications like vaccines, drug delivery, and photo imaging. We propose the possible use of VLPs to study virus-host interaction or internalization.

The feature that makes VLPs important in vaccine development is their high density of epitopes, particulate nature, and lack of genetic material that restricts their replication and makes them safe for the host [26,36,37,38]. In addition to their use in vaccine development, VLPs are also widely investigated to deliver drugs and other small molecules inside the host system. This property is attributed to the internal cavity in VLPs. Several studies also showed the feasibility of using VLPs in photo imaging [37,38]. Different applications of VLPs are shown in Figure 2.

The high epitope density and particulate nature of VLPs make them ideal systems for mounting immune responses. It is essential to mention that VLPs can mount both humoral and cellular immune responses [39,40,41,42,43,44]. Because of their natural resemblance to viruses, VLPs act as pathogen-associated structural patterns (PASP) and are easily recognized and taken up by host immune cells [39,40]. Additionally, their structural properties help activate immune cells like dendritic cells [45]. Due to their ability to mount both humoral and cellular immune responses, VLPs appear to be a better choice for vaccine delivery than purified proteins [46].

**Figure 3:**
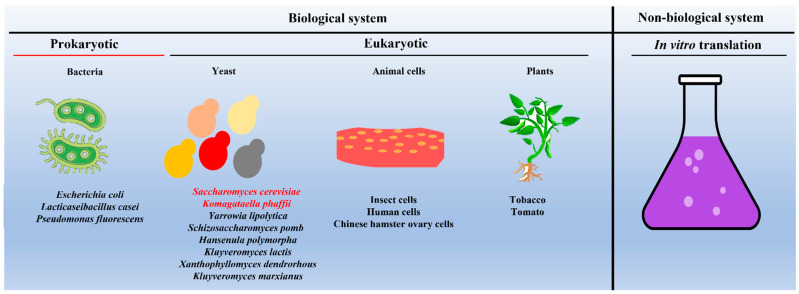
Schematic showing different systems used as hosts for expressing and purifying VLPs. Note: *Saccharomyces cerevisiae* and *Komagataella phaffii* (in red font) remain the most commonly used yeast species. In the case of in vitro production of VLPs, one can use the protein translational machinery of either bacteria (prokaryotes) or yeast (eukaryotes).

Due to their importance in vaccine development and drug delivery, efforts were made to express and purify VLPs from different biological systems and identify the most suitable hosts for producing VLPs commercially. To this end, VLPs are successfully expressed and purified from both the prokaryotic system (for example, *Escherichia coli*) [34] as well as from the eukaryotic system, including yeast (for example, *S. cerevisiae*) [47], insect cell lines [48,49], mammalian cell lines [39,50], and plants [51] (as shown in Figure 3).

Apart from whole cells, several studies have shown that VLPs can be produced by in vitro protein translation systems [52]. The advantages and disadvantages of in vitro protein translation-based VLP generation are discussed by others [22,52,53]. In Table 1, the advantages and disadvantages of different systems used for the expression and purification of VLPs are compared.

## 3. Yeast, Host to Produce VLPs on a Commercial Scale

As mentioned above, several biological systems have been evaluated over the years for a suitable host to produce VLPs on a commercial scale. An ideal host for the commercial purification of VLPs should be nonpathogenic, easy to handle, able to grow on economic media, able to express the protein of interest in the maximum amount, allow proper folding of a large amount of expressed protein, be genetically responsive, and be easily scaled up to industrial measures [54]. The secretion of the expressed protein(s) and VLPs (both enveloped and non-enveloped) into the medium will be another beneficial facet. Looking at these attributes, a yeast-based system appears as an ideal host for the commercial production of VLPs. In Table 1, the advantages, and disadvantages of yeast over other hosts are compared.

In the past, several yeasts were successfully used to express and purify clinically relevant proteins. Yeast species, *S. cerevisiae* and *K. phaffii* (formerly known as *Pichia pastoris*), fall under GARS (Generally Recognized as Safe), which is another significant advantage of using yeast-based systems for the development of VLPs [55]. Unlike the bacterial system, the yeast-based system does not suffer from endotoxin problems [56], and the solubility of expressed proteins and folding are much better in yeast compared to bacteria [57]. Furthermore, utilizing mammalian and insect cells for VLP production is expensive due to the high cost of media and poor scalability [58]. In contrast, yeast can quickly grow on simple media [59]. A yeast species, *K. phaffii*, can be grown to a high cell density on a commercial scale, which is not feasible with animal cells. The rapid growth of yeast cells (unlike animal cells, yeast cells grow faster with a doubling time of around 90–120 min, whereas animal cells have a doubling time of 16–18 h or more) is another advantage [60]. The growth of plants is slow and may take several weeks, months, or even years to reach the desired maturity. Another issue is the varying expression levels in different organs or tissues and more batch-to-batch variation. Other concerns include the possibility of escape into the natural environment [61]. The seasonal variation may severely impact plant growth, so the expression of the protein of interest remains an important consideration. Due to several advantages (as mentioned), yeast has been extensively used for expressing and purifying VLPs, especially *S. cerevisiae* (Table 2) and *K. phaffii* (Table 3).

Apart from the advantages mentioned above, yeast offers several other benefits, including a fully sequenced genome, the availability of vast genetic information, and molecular tools (in terms of vectors, promoters, and markers). Yeasts such as *S. cerevisiae* and *K. phaffii* are accessible and responsive in terms of genetic manipulation. Technology for introducing foreign genes is well established and therefore allows integration of the gene of interest at a precise location in the genome, contrary to animal or plant cells [60]. Under certain conditions, several copies of a gene can be introduced in the same cell, thereby increasing the yield per cell [122]. The yeast-based system also gives the option of constitutive and inducive expression. Compared to plant or animal cells, screening positive clones with a high yield is relatively fast and more economical in yeast [122]. Although in most of the studies, proteins of interest were expressed intracellularly, on several occasions, a protein expressed for VLP formation also gets secreted in culture media; this is another advantage as it is relatively easier to purify proteins from growth media [54]. Apart from *S. cerevisiae* and *K. phaffii*, several other yeast species have been tested for expression and purification of VLPs. (See Table 4 and Table 5).

**Table 3:** Studies where *K. phaffii* yeast was used to generate VLPs.

S. No	Protein Antigen	Virus	Protein Localization	Promoter	References
1	Capsid protein	Red-spotted grouper nervous necrosis virus	IC	Pw42-2	[123]
2	ZS and S	Zika virus	IC	AOX1	[124]
3	112-608aa of the ORF2	Hepatitis E virus	EC	AOX1	[125]
4	P1 and CD3	Poliovirus type I	IC	AOX1	[126,127]
5	Chimeric HPV-HIV L1P18 protein	HPV and HIV	IC	GAP	[128]
6	NY-ESO-1 cancer testis antigen	Norovirus	EC	AOX1	[129]
7	Surface antigen	Hepatitis C virus	IC	AOX1	[130,131]
8	P1 and 3CD	Enterovirus 71	EC	AOX1	[132,133]
9	E domain III	Dengue Virus	IC	AOX1	[134,135]
10	P1 and 3CD	Coxsackievirus A16	EC	GAP	[136]
11	VP1	Norovirus	EC	AOX1	[137]
12	E antigen	Dengue virus	IC	AOX1	[138,139,140,141,142,143]
13	prME	Japanese encephalitis virus	EC	AOX1	[144]
14	E antigen	Dengue virus	EC	GAP	[145]
15	Matrix protein	Nipah virus	IC	AOX1	[146]
16	P1 and 3CD	Enterovirus D68	IC	AOX1	[147]
17	prM and E protein	Tick-borne encephalitis virus	EC	GAP	[148]
18	P1 and 3CD	Coxsackievirus A10	IC	AOX1	[149]
19	Surface antigen	Hepatitis B virus	IC	AOX1	[150,151,152,153,154,155,156]
20	prM and E protein	Dengue virus	IC	GAP	[157]
21	L1	HPV16 and 18	IC	AOX1	[158,159,160]
22	L1	HPV 52		AOX1	[161]
23	Capsid protein	Cowpea chlorotic mottle virus	EC	AOX1	[162]
24	L1	HPV 58	IC	AOX1	[163]
25	Envelope protein domain III (EDIII), hepatitis B surface antigen	Dengue virus	IC	AOX1	[164]
26	VP2	Infectious bursal disease virus	IC	AOX1	[165]
27	P1 and 3CD	Coxsackievirus A16	IC	AOX1	[166]
28	Core protein	Hepatitis B virus	IC	AOX1	[167,168]
29	E protein	Dengue virus	IC	AOX1	[169]
30	L2	Grapevine fanleaf virus	EC	AOX1	[170]
31	Capsid protein (VP60)	Rabbit hemorrhagic disease virus	IC	AOX1	[171]
32	HBc-influenza virus LAH domain	Hepatitis B/Influenza H3N2 virus	IC	AOX1	[172]
33	CoreE1E2 Protein	Hepatitis C virus	EC	AOX1	[173]
34	P1 and 3CD	Coxsackievirus A6	IC	AOX1	[174]
35	L1, L2	HPV 16	IC	AOX1	[175]
36	prM/Env	Japanese encephalitis virus	IC	AOX1	[176]
37	Den2E-HBsAg	Dengue/Hepatitis B virus	IC	AOX1	[177]
38	prM and E protein	Dengue virus	IC	GAP	[178]
39	Polyprotein	Chikungunya virus	EC	AOX1	[179]
40	L1	HPV 16			[180]
41	Major capsid protein	Iridovirus	EC	AOX1	[181]
42	Surface antigen	Hepatitis B virus			[182]
43	VP1	Rabbit hemorrhagic disease virus	IC	AOX1	[183]
44	L1	Bovine papillomavirus 1,2,4			[184]
45	L1	HPV 16	IC	AOX1	[185]
46	Capsid protein	Norovirus	IC	AOX1	[186]
47	L1	HPV 16	EC	PGK1	[187]
48	Core protein	Hepatitis B virus			[188]
49	VP1	Calicivirus virus	EC	AOX1	[189]
50	Core protein	Hepatitis C virus	IC	AOX1	[190,191,192,193]

IC: intracellular; EC: extracellular.

## 4. Bottleneck in the Use of a Yeast-Based System on a Commercial Scale

In the previous section, we discussed some salient features that make yeast a suitable host for the commercial production of VLPs. Indeed, many commercially available vaccines are produced using yeast (discussed in detail in the next section). Despite this, several issues limit the use of the yeast-based system for commercial expression and purification of VLPs. In this section, we will point out the reasons that hamper the full utilization of yeast-based platforms for the commercial production of VLPs. One of the critical issues with using yeast to produce proteins (which will be used as vaccines) is the inability of yeast cells to perform protein glycosylation like humans [207].

Further, proteins in yeast cells are, in general, more heavily glycosylated compared to animal cells, including human cells [208]. Additionally, the glycosylation pattern differs in humans and yeast, which may have significant implications in terms of the antigenicity, solubility, and stability of proteins, especially when injected into the body [209]. However, this issue can be addressed to a certain extent by treating purified proteins with deglycosylase enzymes or modifying the primary amino acid sequence where possible. Apart from this, efforts are made to engineer yeast cells (through pathway modification) to minimize the problem of heavy mannosylation of proteins. For example, the glycoengineered strain of *K. phaffii* from Biogrammatics showed a significant reduction in glycosylation of expressed proteins (https://www.biogrammatics.com (accessed on 17 December 2022).

Another issue limiting the use of yeast for producing VLPs is that many viruses are enveloped. To date, only one study has shown the possibility of producing enveloped VLPs (eVLPs) using yeast as a host [210]. The success of producing eVLPs using the yeast-based system on an industrial scale remains to be determined. Indeed, this is where animal cells have advantages over yeast-based systems; however, future research can help manipulate cell membranes and may open the way for the secretion of eVLPs in yeast.

## 5. In Vivo VLP Assembly and Secretion

Secretion of fully assembled VLPs into culture media is desirable as it enables an economical and straightforward purification of VLPs from the host system. So far, insects and mammalian cells are known to secrete fully assembled VLPs into culture media [45,211]. This feature is especially important when one is working with eVLPs. However, the high media cost and level to which scale-up is possible with animal cell lines remain significant concerns (reviewed in [212]). *S. cerevisiae* and *K. phaffii* are known to grow on cheap media and can grow well in big industrial vessels (especially *K. phaffii*); however, the secretion of fully assembled VLPs into culture media is possible in a few cases. (Table 2, Table 3, Table 4 and Table 5). Even before secretion, it is important to see whether VLPs can be assembled within yeast cells in the cytoplasm. Several studies in the past have already shown the in vivo assembly of VLPs followed by their secretion [129,144,145,148,179,210]. Again, this showed the advantage of yeast over the bacterial system. Based on published studies, some VLPs get secreted into the media while others are not (Table 2, Table 3, Table 4 and Table 5). Secretion of VLPs depends upon the proteins used for VLP generation and VLP size. Therefore, one must check the possibility of secretion of VLPs for each virus and protein used.

## 6. Aggregation of VLPs, a Matter of Concern

The production of VLPs on a commercial scale using the different hosts mentioned above is now becoming common practice. Several companies dealing in vaccines or drugs are producing VLPs commercially. Almost all VLP production suffers from the common issue of VLP aggregation when stored at a low-salt concentration and at 2–8 °C [213,214,215,216,217]. Dynamic Light Scattering (DLS), Transmission Electron Microscopes (TEM), and Atomic Force Microscopes (AFM) can detect the aggregation of VLPs, approaches commonly used for VLP characterization (reviewed by [28]). In addition, turbidity during elution or storage can also inform about possible VLP aggregation. The main problem with the aggregation of VLPs is that it affects immunogenicity and the level of an immune response. Aggregation of VLPs reduces their recovery using POROS resin used for the purification of VLPs on a commercial scale [111]. It was observed that the immune response raised by aggregated VLPs is lower than that raised by non-aggregated VLPs [213,218,219,220,221]. Additionally, aggregation can affect dose formulation. To obtain highly monodisperse VLPs, the manufacturer performs an ultracentrifuge or size exclusion to separate clean and aggregated VLPs. However, this leads to considerable wastage of VLPs and reduces the recovery of useful VLPs.

To prevent or minimize the aggregation of VLPs, different approaches have been used in the past. For example, adding L-arginine and glycine improves VLP recovery [221,222]. Sugars like sorbitol and trehalose are also shown to solubilize and improve VLP recovery by preventing their aggregation and by solubilizing protein monomers [210,212]. The use of polyethylene glycol (PEG) or glycerol also improves the solubilization of VLPs [223,224]. Apart from this, using surfactants or emulsifiers like Tween-80 is also helpful in preventing the aggregation of VLPs and improving the recovery of VLPs [225]. In several published studies, the author has added these compounds at one or another step in the purification of VLPs. Sometimes changes in the pH of the buffer and the buffer composition were also found helpful in preventing the aggregation of VLPs [226]. Additionally, to obtain VLPs of a uniform size, in vitro disassembly followed by reassembly is recommended [227]. Therefore, it requires substantial effort to find suitable conditions that improve the recovery of VLPs while simultaneously preventing their aggregation.

## 7. Success Story

In previous sections, we provided a descriptive background of VLPs, various hosts available for their production, issues, and the suitability of yeast to produce VLPs. We also mentioned one of the most common problems encountered in the purification of VLPs. Now is the time to discuss some cases where VLPs have been used successfully as a vaccine or drug delivery system. Although we will mainly focus on VLPs produced using yeast that are currently on the market for public use, it is essential to mention that VLPs purified from other systems are also floating on the market [22]. 

The first VLP-based vaccine to hit the market was Gardasil, followed by Gardasil9; both products were branded under Merck and were against HPV-induced cancer. GlaxoSmithKline introduced another VLP-based vaccine (Cervarix) against HPV. It is important to note that Cervarix was effective against both the HPV16 and HPV18 viral strains. Cervarix from GlaxoSmithKline was taken off the market (USA) in 2016, while Gardasil and Gardasil9 are still available for public use. Notably, Gardasil and Gardasil9 are from yeast, while Cervarix is from the Baculovirus expression system, which uses High Five Rix4446 cells derived from the insect *Trichoplusia ni.* Apart from the two vaccines (from yeast) mentioned, another vaccine, Mosquirix against *Plasmodium falciparum*, responsible for malaria, is commercially approved [228].

Above, we mentioned the VLP-based vaccines currently in public use. Apart from this, several VLP-based vaccines are under clinical trials. An example vaccine against HIV is under clinical trial [43]. Apart from yeast-derived VLPs, several VLP-based vaccines derived from non-yeast hosts are also in public use or clinical trials [22]. Table 6 compiles the list of VLPs produced in yeast currently in use and those in different phases of clinical trials.

## 8. Conclusions and Future Direction

Based on the points discussed above, during the last few years, VLPs have gained significant importance and acceptance as a means of vaccine and drug delivery. Apart from their use in a vaccine, VLPs are also noted for their potential use in drug delivery (chemical salts or small molecules) as well as for the delivery of nucleic acids (DNA/RNA) [22,229,230,231,232,233]. This approach to drug delivery offers the advantage of being biodegradable (unlike metal-based nanoparticles). Several studies from different labs have already showed proof of concept where VLPs were used to deliver drugs and nucleic acids [22]. Because they resemble viruses, VLPs can be used to study virus-host interactions and virus internalization by host cells. This aspect can be a game changer as it will eliminate or minimize the use of handling infectious entities. Since VLPs can be obtained from companies, these can be ordered as standard reagents (like antibodies), which can speed up research activity (Figure 2). For example, Advance ImmunoChemical Inn supplies HPV18 L1 VLPs (https://www.advimmuno.com).

Interestingly, more and more VLPs are now expressed in yeast. Apart from *S. cerevisiae* and *K. phaffii,* other yeast species, including *H. polymorpha*, species of *Kluyveromyces, Y. lipolytica, X. dendrorhous,* and *S. pombe*, have also been used for VLP expression and purification. Additionally, over the years, more and more studies have appeared in which *K. phaffii* was used to produce VLPs. Although eVLPs have yet to be produced using yeast (on a commercial scale), this remains one of the concerns with using yeast for VLP production. Since considerable effort and resources are used during the purification of VLPs, the secretion of fully assembled VLPs into culture media is ideal, making the purification of VLPs economical. The secretion of VLPs is common in the case of animal cells, but in the case of yeast, only a few studies are available where VLPs have been successfully secreted into media [64,125,144,145,189]. Although yeast species other than *S. cerevisiae* and *K. phaffii* are also used for producing VLPs, whether using those species on an industrial scale is feasible remains to be determined. VLPs purified from *S. cerevisiae* are already approved by the FDA and are currently in public use. It will be interesting to see when we will have VLPs purified from *K. phaffii*, which is FDA-approved and available for public use. It is anticipated that we may soon have a VLP-based vaccine where *K. phaffii* is used as a host. Preclinical evaluation of HBc VLPs (purified from *K. phaffii*) against hepatocellular carcinoma is underway [234,235]. This is important as more and more VLPs are purified using *K. phaffii*. Since *K. phaffii* can grow to high cell densities on cheap media, the use of VLPs purified from *K. phaffii* may also reduce the cost of VLP-based vaccines.

On a commercial scale, the purification of VLPs uses a chromatography column (such as POROS HS50). These columns get clogged by yeast lipids (due to the irreversible binding of lipids to resin) and lipid droplets. Therefore, to prevent or minimize any such issue, it is essential to use yeast strains that do not form lipid droplets [236]. In most of the studies using *K. phaffii*, the authors used the GS115 strain. However, using protease-deficient strains (for example, SMD1163) to minimize the degradation of an expressed protein is recommended [24]. Intracellular degradation of an expressed protein can affect the assembly and recovery of VLPs.

In the end, several hosts are available for the production of VLPs, each offering some advantages over the others. Certainly, yeast has some significant benefits as a host for the commercial production of VLPs compared to other available hosts. However, the inability of yeast to secrete VLPs of important viruses remains a critical bottleneck. Therefore, more work in strain engineering will be needed to fully tap the potential of yeast for the production of VLPs. It may involve host engineering, antigen engineering, modification of culture media, and buffers, thus representing several channelings and opportunities.

## Figures and Tables

**Table 1:** A comparison of the different model systems used as a host for the production of VLPs [22,54].

Feature	Bacteria	Insect Cells	Mammalian Cells	Plant	Yeast
Production cost	Low	High	High	Moderate	Low
Growth media	Simple	Complex	Complex	Simple	Simple
Growth	Fast	Slow	Slow	Very slow	Fast
Growth duration	Very small	Small	Small	Long	Small
Indoor/Outdoor	Indoor	Indoor	Indoor	Outdoor/polyhouse	Indoor
Scale-up	Easy	Very difficult	Very difficult	Difficult	Easy
Secretion	No	Yes	Yes	NA	Yes
Enveloped/non-enveloped	Non-enveloped	Enveloped/Non-enveloped	Enveloped/Non-enveloped	NA	Non-enveloped/Enveloped possible
Speed of transformant screening	Very fast	Slow	Slow	Very slow	Fast
Effect of seasonal variations	No	No	No	Yes	No

NA: clear information not available.

**Table 2:** Studies in which *S. cerevisiae* yeast was used to generate VLPs.

S. No	Protein Antigen	Virus	Protein Localization	Promoter	References
1	VP2	Human parvovirus 4	IC	Hybrid GAL10-PYK1promoter	[62]
2	Capsid protein	Hepatitis E virus	IC	GAL promoter	[63]
3	Capsid protein	Porcine circovirus type 2	EC	GPD, TEF2	[64]
4	Nucleocapsid protein	Sendai virus	IC	GAL7	[65]
5	VP2, VP1	Human bocaviruses	IC		[66]
6	Surface antigen	Hepatitis B virus	IC	GAL	[67,68,69]
7	p55(gag)	HIV-1	EC		[70]
8	VP1	Human polyomaviruses	IC	GAL	[71]
9	L1	HPV 16	IC	GAL10	[72,73]
10	Nucleocapsid protein	Tioman virus	IC	GAL10	[74]
11	L-HDAg and surface antigen	Hepatitis delta virus	IC	GAD	[75]
12	Capsid protein	Porcine circovirus type 2	IC	GAL10	[76]
13	Capsid protein	Enterovirus 71	IC	GAL10	[77]
14	VP1	Human and non-human polyomaviruses	IC	GAL	[78]
15	Capsid protein	Adeno-associated virus	IC	GAL1	[79]
16	Nucleocapsid protein	Human parainfluenza virus 4	IC	GAL7	[80]
17 *	Coat protein	Cacteriophage Qbeta virus	IC	GAL	[81]
18	Capsid protein	Nervous necrosis virus	IC	GAL	[82]
19	VP1,2	Parvovirus B19	IC	ADH2/GAPDH	[83,84]
20	VP1	Bird polyomaviruses	IC	GAL	[85]
21	P1	Enterovirus 71 and Coxsackievirus A16	IC	GAL1	[86]
22	Capsid protein	Porcine circovirus type 2	IC	GAL1	[87]
23	VP2,6,7	Rotavirus	IC	PGK1, TEF1	[88,89]
24	VP2	Human parvovirus 4	IC	GAL1-10	[90]
25	Nucleocapsid protein	Human parainfluenza virus 2	IC	GAL	[91]
26	Nucleocapsid protein	Menangle virus	IC	GAL7	[92]
27	Gag	HIV-1	IC	GAP	[93]
28	VP2	Porcine parvovirus	IC	GAL1-10	[94]
29	P1, CD3	Coxsackievirus A16	IC	GAL1	[95]
30	Capsid protein	Porcine circovirus type 2	IC	GAL	[96]
31	VP1,2	Hepatitis B/Polyomavirus	IC	GAL7	[97]
32	VP1	Hamster polyomavirus	IC		[98]
33	L1/L1 + L2	Cottontail rabbit papillomavirus	IC	GAL1-10	[99]
34	L1	HPV 11	IC	GAL	[100,101,102]
35	Coat protein	Potyvirus (Johnsongrass mosaic virus)	IC	ADC1	[103]
36	P1, CD3	Poliovirus type I	IC		[104]
37		HIV-1	IC		[105]
38	L1	HPV 16	IC	GAL	[106]
39	L1	HPV 6,11 16	IC	GAL	[107]
40	VP1 with Puumala hantavirus nucleocapsid protein segments	Hamster polyomavirus	IC	Hybrid GAL10-PYK1	[108]
41	M protein	Hepatitis B virus	IC	GAL10/CYC1	[109]
42	VP1,2	Goose hemorrhagic polyomavirus	IC	GAL	[110]
43	CEA/VP1	Hamster polyomavirus	IC	GAL	[111]
44	E7 oncoprotein of HPV16	Hepatitis B virus	IC		[112]
45	Capsid protein	Red-spotted grouper nervous necrosis virus	IC	GAL10	[113]
46	L1	HPV 58	IC	GAL10	[114]
47	C69R variant of surface antigen	Hepatitis B virus	IC	GAL10/CYC1	[115]
48	VP1	Human polyomavirus 2	IC	GAL	[116]
49	VP2	Parvovirus B19	IC	GAL1	[117]
50	L1	HPV 11	IC	GAL110-11	[118]
51	VP1 with pre-S1 region of the Hepatitis B virus	Hamster polyomavirus	IC	GAL	[119]
52	Surface antigen	Hepatitis B virus	IC	GAL10	[120]
53	L1	HPV 16	IC		[121]

* Additionally, expressed in *K. phaffii*; IC: intracellular; EC: extracellular.

**Table 4:** Studies where *H. polymorpha* yeast was used to generate VLPs.

S. No	Protein Antigen	Disease	Secretion Localization	Promoter	References
1	Porcine circovirus type 2b capsid protein	Postweaning multisystemic wasting disease (PMWS) in pigs	IC	MOX (methanol)	[194]
2	Bovine viral diarrhea virus (BVDV) glycoprotein E2	Bovine diarrhea	IC	FMD (formate dehydrogenase)	[195]
3	Membrane integral small surface protein (dS) of the duck Hepatitis B virus	Bovine diarrhea	IC	MOX (methanol)	[196]
4	Surface antigen	Hepatitis E	IC		[197]
5	Sporozoite antigen	Malaria	IC		[198]
6	Sporozoite antigen	Malaria	IC	MOX (methanol)	[199]
7	Glycoprotein E2 ectodomain	Hepatitis C	EC		[200]

IC: intracellular; EC: extracellular.

**Table 5:** Studies where yeast species (other than those in Table 2, Table 3 and Table 4) were used to generate VLPs.

S. No	Protein Antigen	Disease	Secretion Localization	Yeast Species	References
1	Capsid protein of porcine circovirus	Postweaning multisystemic wasting syndrome (PMWS) and porcine circovirus diseases (PCVDs)	IC	*Kluyveromyces marxianus*	[201]
2	VP1 of murine polyomavirus	Cancer	IC	*Kluyveromyces lactis*	[202]
3	VP2 of porcine parvovirus	Embryonic and fetal loss, death, and mummification	IC	*Kluyveromyces marxianus*	[203]
4	Capsid protein of red-spotted grouper nervous necrosis virus	Viral encephalopathy and retinopathy	IC	*Yarrowia lipolytica*	[204]
5	Totivirus capsid protein	NA	IC	*Xanthophyllomyces dendrorhous*	[205]
6	Tobacco mosaic virus coat protein	Mosaic-like mottling and discoloration on the leaves	IC	*Schizosaccharomyces pombe*	[206]

IC: intracellular; EC: extracellular. NA: not applicable as no disease is reported where Totivirus is responsible.

**Table 6:** VLP-based vaccines expressed in yeast that are either approved by the FDA for commercial use or are undergoing clinical trials.

Trade Name	Infectious Agent	Disease	Company	Antigen	Status
Gardasil^®^	HPV	Cervical carcinoma	Merck	L1	Approved
Gardasil9^®^	HPV	Cervical carcinoma	Merck	L1	Approved
Mosquirix™	*P. falciparum*	Malaria	Univ. of Rochester AVEG, Rochester, New York, United States	*P. falciparum* circumsporozoite protein fused to the Hepatitis B surface antigen	Approved
NA	HIV	Acquired immune deficiency syndrome		HIV p17/p24: Ty-VLP	Clinical trial Phase 1(Clinical trial No.: NCT00001053) [43]
Hepavax-Gene	HBV	Hepatocellular carcinoma	Crucell (Dusseldorf-Germany)	SHBs, MHBs	Licensed *
Fendrix	HBV	Hepatocellular carcinoma	GSK (Belgium)	SHBs	Licensed
Heplisav-B	HBV	Hepatocellular carcinoma	Dynavax (Oakland-USA)	SHBs	Licensed *
Engerix	HBV	Hepatocellular carcinoma	GSK (UK)	SHBs	Licensed
Recombivax HB (H-B-Vac^®^-II)	HBV	Hepatocellular carcinoma	Merck Vaccine (Canada)	SHBs	Licensed

NA: Not Available; HPV: *Human Papillomavirus* [22]. * *H. polymorpha* yeast was used as the expression host, while in the rest of the cases, *S. cerevisiae* was used. HBV: Hepatitis B virus.

## Data Availability

Not applicable.
